# Supplementary figures and images for: Global sex differences in hygiene norms and their relation to sex equality
Source: PLOS Glob Public Health. 2022 Jun 21;2(6):e0000591. doi: 10.1371/journal.pgph.0000591 (PMC10021886; doi:10.1371/journal.pgph.0000591)

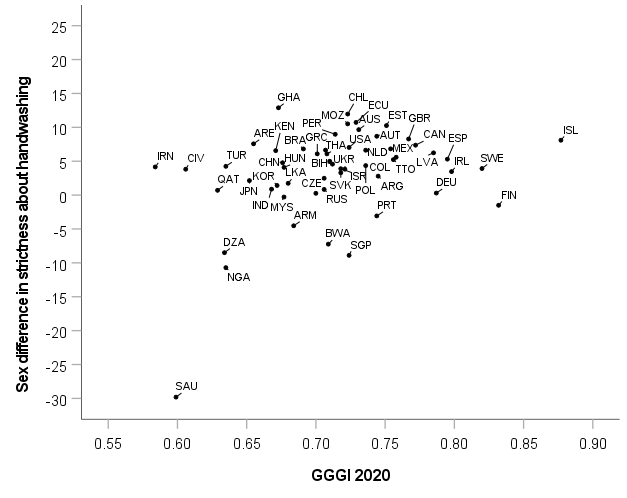

Supplement: S1 Fig — GGGI data for 56 countries taken from the World Economic Forum’s Global Gender Gap Report for 2020. Labels are ISO country codes. (TIF) [file pgph.0000591.s001.tif]

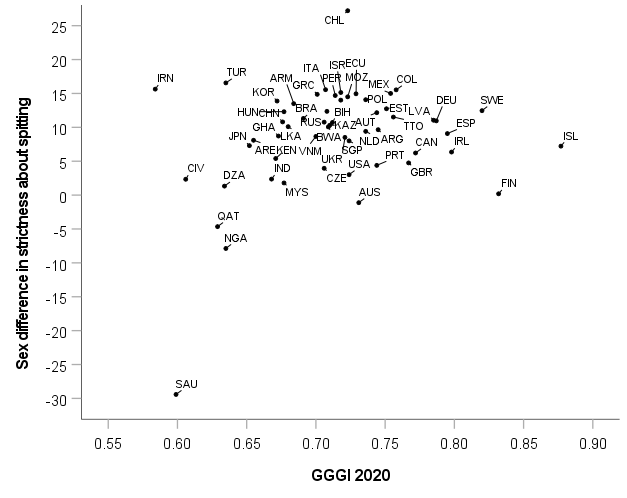

Supplement: S2 Fig — GGGI data for 56 countries taken from the World Economic Forum’s Global Gender Gap Report for 2020. Labels are ISO country codes. (TIF) [file pgph.0000591.s002.tif]
