# Supplementary material for: Global sex differences in hygiene norms and their relation to sex equality
Source: PLOS Glob Public Health. 2022 Jun 21;2(6):e0000591. doi: 10.1371/journal.pgph.0000591 (PMC10021886; doi:10.1371/journal.pgph.0000591)
Supplement: S1 Table — (DOCX) [file pgph.0000591.s003.docx]

**S1 Table.** **Sample characteristics per country.**

| Country | Sample size | Mean age, in years | % women | % students |
| --- | --- | --- | --- | --- |
| Algeria | 70 | 23 | 79 | 100 |
| Argentina | 448 | 26 | 69 | 47 |
| Armenia | 349 | 24 | 67 | 67 |
| Australia | 247 | 20 | 76 | 100 |
| Austria | 341 | 22 | 67 | 100 |
| Bosnia and Herzegovina | 243 | 21 | 50 | 100 |
| Botswana | 45 | 21 | 82 | 100 |
| Brazil | 281 | 31 | 55 | 69 |
| Canada | 425 | 20 | 67 | 100 |
| Chile | 129 | 21 | 43 | 100 |
| China | 1009 | 22 | 71 | 80 |
| Colombia | 342 | 26 | 61 | 67 |
| Czech Republic | 378 | 28 | 77 | 69 |
| Ecuador | 282 | 23 | 62 | 85 |
| Estonia | 341 | 30 | 79 | 65 |
| Finland | 235 | 31 | 82 | 89 |
| Germany | 656 | 29 | 69 | 30 |
| Ghana | 276 | 23 | 57 | 78 |
| Greece | 506 | 27 | 69 | 73 |
| Hungary | 448 | 25 | 80 | 79 |
| Iceland | 469 | 31 | 77 | 78 |
| India | 261 | 20 | 88 | 96 |
| Iran | 222 | 21 | 72 | 100 |
| Ireland | 238 | 22 | 58 | 95 |
| Israel | 334 | 28 | 60 | 68 |
| Italy | 386 | 23 | 59 | 100 |
| Ivory Coast | 189 | 26 | 43 | 100 |
| Japan | 557 | 20 | 53 | 100 |
| Kazakhstan | 186 | 20 | 60 | 100 |
| Kenya | 191 | 22 | 49 | 100 |
| Latvia | 332 | 30 | 72 | 89 |
| Malaysia | 339 | 25 | 53 | 69 |
| Mexico | 141 | 25 | 70 | 100 |
| Mozambique | 203 | 23 | 35 | 100 |
| Netherlands | 270 | 22 | 55 | 100 |
| Nigeria | 298 | 28 | 57 | 61 |
| Peru | 257 | 34 | 68 | 41 |
| Poland | 543 | 35 | 71 | 44 |
| Portugal | 140 | 24 | 89 | 100 |
| Qatar | 64 | 29 | 88 | 42 |
| Russia | 382 | 23 | 77 | 100 |
| Saudi Arabia | 302 | 25 | 25 | 71 |
| Singapore | 201 | 22 | 69 | 100 |
| Slovakia | 356 | 30 | 56 | 66 |
| South Korea | 362 | 27 | 55 | 65 |
| Spain | 253 | 30 | 43 | 51 |
| Sri Lanka | 189 | 23 | 62 | 100 |
| Sweden | 203 | 27 | 54 | 100 |
| Thailand | 203 | 20 | 70 | 100 |
| Trinidad and Tobago | 194 | 26 | 80 | 65 |
| Turkey | 229 | 22 | 83 | 100 |
| United Arab Emirates | 302 | 20 | 64 | 100 |
| Ukraine | 248 | 29 | 65 | 59 |
| United Kingdom | 425 | 24 | 84 | 71 |
| United States | 658 | 20 | 77 | 100 |
