# Supplementary material for: Global sex differences in hygiene norms and their relation to sex equality
Source: PLOS Glob Public Health. 2022 Jun 21;2(6):e0000591. doi: 10.1371/journal.pgph.0000591 (PMC10021886; doi:10.1371/journal.pgph.0000591)
Supplement: S2 Table — (DOCX) [file pgph.0000591.s004.docx]

**S2 Table. Mixed-level analyses of the strictness about handwashing and spitting using the Global Gender Gap Index (GGGI) as a measure of sex equality.**

|  | Strictness about handwashing | | | | Strictness about spitting | | | |
| --- | --- | --- | --- | --- | --- | --- | --- | --- |
|  | Countries w. *below* average GGGI | | Countries w. *above* average GGGI | | Countries w. *below* average GGGI | | Countries w. *above* average GGGI | |
| Variable | w/o cont. | w. cont | w/o cont. | w. cont | w/o cont. | w. cont | w/o cont. | w. cont |
| Female | 1.8 | 1.0 | 5.6 ^a^ | 4.8 ^a^ | 7.7 ^a^ | 7.2 ^a^ | 10.4 ^a^ | 9.7 ^a^ |
| Female ✕ GGGI | 76 ^d^ | 89 ^d^ | -12 | -8 | 113 ^b^ | 127 ^b^ | -44 ^d^ | -39 ^d^ |
| N (individuals) | 8721 | 8721 | 8911 | 8911 | 8721 | 8721 | 8911 | 8911 |
| N (countries) | 28 | 28 | 28 | 28 | 28 | 28 | 28 | 28 |
| BIC | 78535 | 78065 | 79990 | 79729 | 80983 | 83154 | 80786 | 83021 |

Results from analysis strictness about handwashing and spitting using linear mixed-effect models. Note: Entries are unstandardized coefficients. Models “without controls” included intercept and sex equality. Models “with controls” additionally included age, dummy for student, response style, perceived threat of disease and valuation of self-control (the latter two variables both at the individual level and aggregated to the country level). GGGI data are taken from the World Economic Forum’s Global Gender Gap Report for 2020.

^a^: *p* < .001

^b^: *p* < .01

^d^: *p* < .10
