# Supplementary material for: Global sex differences in hygiene norms and their relation to sex equality
Source: PLOS Glob Public Health. 2022 Jun 21;2(6):e0000591. doi: 10.1371/journal.pgph.0000591 (PMC10021886; doi:10.1371/journal.pgph.0000591)
Supplement: S1 File — (DOCX) [file pgph.0000591.s005.docx]

International study of meta-norms - Final MASTER

Introduction1 Description: You are invited to participate in an international study of social norms. You will be asked a set of questions related to social norms. These include questions about some animations that will be shown to you.

Introduction2 **Risks and Benefits:** There are no risks to participation other than what you would encounter in daily life. You do not have to answer any question you do not want to. Please note that there are no right or wrong answers, and we hope that you will respond with whatever most immediately comes to mind. By participating you will have the opportunity to learn about how this research is conducted and contribute to cross-cultural understanding of social norms.

Introduction3 **Time involvement:** Your participation in this experiment should take approximately 30 minutes.

Introduction4 **How will this data be used?** This study collects data from thousands of participants across the globe. The dataset will be analyzed, and findings will be used for publication in scientific journals. The dataset will be stored indefinitely and shared with other researchers to be used for research and teaching purposes. However, the data you provide will be anonymous, that is, you will not be asked for any information by which your responses can be traced back to you. Therefore, nobody but you will know how you individually responded.

Intruduction5 **Your Rights:** If you have read this information and have decided to participate in this project, please understand that your participation is voluntary and that you have the right to discontinue participation at any time without penalty. You have the right to refuse to answer any questions you prefer not to respond to. Your individual privacy will be maintained in all published and written data resulting from the study.

Consent **Your decision:** If you would like to participate, please mark the box to indicate your decision:

- I agree to participate and consent to the data being used as described above. (4)

End of Block: Introduction

Start of Block: Hofstede

Hofstede_info Please think of an ideal job, disregarding your present job, if you have one. In choosing an ideal job, how important would it be to you to...

| 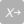 |
| --- |

Individualism_Q1 ...have sufficient time for your personal or home life?

- of utmost importance (5)
- very important (4)
- of moderate importance (3)
- of little importance (2)
- of very little or no importance (1)

| 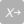 |
| --- |

PowerDistance_Q2 ...have a boss (direct superior) you can respect?

- of utmost importance (5)
- very important (4)
- of moderate importance (3)
- of little importance (2)
- of very little or no importance (1)

| 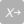 |
| --- |

Individualism_Q4 ...have security of employment?

- of utmost importance (1)
- very important (2)
- of moderate importance (3)
- of little importance (4)
- of very little or no importance (5)

| 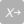 |
| --- |

Individualism_Q6 ...do work that is interesting?

- of utmost importance (5)
- very important (4)
- of moderate importance (3)
- of little importance (2)
- of very little or no importance (1)

| 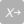 |
| --- |

PowerDistance_Q7 ...be consulted by your boss in decisions involving your work?

- of utmost importance (1)
- very important (2)
- of moderate importance (3)
- of little importance (4)
- of very little or no importance (5)

| 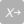 |
| --- |

Individualism_Q9 ...have a job respected by your family and friends?

- of utmost importance (1)
- very important (2)
- of moderate importance (3)
- of little importance (4)
- of very little or no importance (5)

| 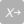 |
| --- |

Indulgence_Q11 In your private life, how important is keeping time free for fun?

- of utmost importance (5)
- very important (4)
- of moderate importance (3)
- of little importance (2)
- of very little or no importance (1)

| 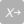 |
| --- |

Indulgence_Q12 In your private life, how important is moderation: having few desires?

- of utmost importance (1)
- very important (2)
- of moderate importance (3)
- of little importance (4)
- of very little or no importance (5)

| 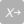 |
| --- |

Indulgence_Q16 Are you a happy person?

- always (5)
- usually (4)
- sometimes (3)
- seldom (2)
- never (1)

| 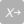 |
| --- |

Indulgence_Q17 Do other people or circumstances ever prevent you from doing what you really want to?

- always (1)
- usually (2)
- sometimes (3)
- seldom (4)
- never (5)

| 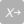 |
| --- |

PowerDistance_Q20 How often, in your experience, are subordinates afraid to contradict their boss (or students their teacher)?

- never (1)
- seldom (2)
- sometimes (3)
- usually (4)
- always (5)

| 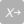 |
| --- |

PowerDistance_Q23 To what extent do you agree or disagree with: An organization structure in which certain subordinates have two bosses should be avoided at all cost

- strongly agree (5)
- agree (4)
- undecided (3)
- disagree (2)
- strongly disagree (1)

End of Block: Hofstede

Start of Block: Experience of punishment

| 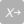 |
| --- |

You_confront How often do you confront someone for doing something inappropriate?

- never (1)
- seldom (2)
- sometimes (3)
- usually (4)
- always (5)

| 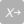 |
| --- |

Confront_you How often does someone confront you for doing something inappropriate?

- never (1)
- seldom (2)
- sometimes (3)
- usually (4)
- always (5)

| 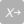 |
| --- |

You_Talk How often do you talk to others about someone who has done something inappropriate?

- never (1)
- seldom (2)
- sometimes (3)
- usually (4)
- always (5)

| 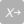 |
| --- |

Talk_about_you How often do you experience that someone has been talking about you doing something inappropriate?

- never (1)
- seldom (2)
- sometimes (3)
- usually (4)
- always (5)

| 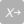 |
| --- |

You_avoid How often do you avoid someone who has done something inappropriate?

- never (1)
- seldom (2)
- sometimes (3)
- usually (4)
- always (5)

| 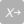 |
| --- |

Avoids_you How often do you experience that someone avoids you when they think you have done something inappropriate?

- never (1)
- seldom (2)
- sometimes (3)
- usually (4)
- always (5)

End of Block: Experience of punishment

Start of Block: WVS

| 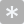 |
| --- |

Important_children Here is a list of qualities that children can be encouraged to learn at home. Which, if any, do you consider to be especially important? Please choose up to five!

- Independence (1)
- Hard work (2)
- Feeling of responsibility (3)
- Imagination (4)
- Tolerance and respect for other people (5)
- Thrift, saving money and things (6)
- Determination, perseverance (7)
- Religious faith (8)
- Unselfishness (9)
- Obedience (10)

Justified_info Please tell us for each of the following actions whether you think it can always be justified, never be justified, or something in between.  (1 = Never justifiable 10 = Always justifiable)

| 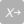 |
| --- |

Just_homo Homosexuality

- 1 = Never justifiable (1)
- 2 (2)
- 3 (3)
- 4 (4)
- 5 (5)
- 6 (6)
- 7 (7)
- 8 (8)
- 9 (9)
- 10 = Always justifiable (10)

| 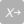 |
| --- |

Just_divorce Divorce

- 1 = Never justifiable (1)
- 2 (2)
- 3 (3)
- 4 (4)
- 5 (5)
- 6 (6)
- 7 (7)
- 8 (8)
- 9 (9)
- 10 = Always justifiable (10)

| 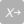 |
| --- |

Just_abortion Abortion

- 1 = Never justifiable (1)
- 2 (2)
- 3 (3)
- 4 (4)
- 5 (5)
- 6 (6)
- 7 (7)
- 8 (8)
- 9 (9)
- 10 = Always justifiable (10)

| 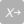 |
| --- |

Just_suicide Suicide

- 1 = Never justifiable (1)
- 2 (2)
- 3 (3)
- 4 (4)
- 5 (5)
- 6 (6)
- 7 (7)
- 8 (8)
- 9 (9)
- 10 = Always justifiable (10)

| 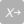 |
| --- |

Just_beatwife For a man to beat his wife

- 1 = Never justifiable (1)
- 2 (2)
- 3 (3)
- 4 (4)
- 5 (5)
- 6 (6)
- 7 (7)
- 8 (8)
- 9 (9)
- 10 = Always justifiable (10)

| 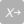 |
| --- |

Just_violence To use violence against other people

- 1 = Never justifiable (1)
- 2 (2)
- 3 (3)
- 4 (4)
- 5 (5)
- 6 (6)
- 7 (7)
- 8 (8)
- 9 (9)
- 10 = Always justifiable (10)

End of Block: WVS

Start of Block: Hygiene norms

| 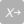 |
| --- |

Hygene_BrushTeeth How often do you think people should brush their teeth?

- three times a day or more (5)
- two times a day (4)
- once a day (3)
- at least once a week (2)
- less than once a week (1)
- never (0)

| 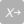 |
| --- |

Hygene_Spit Where do you think it is not appropriate for people to spit? Tick any that apply

- in kitchen sink (1)
- on the sidewalk (2)
- on kitchen floor (3)
- on the soccer field (4)
- in the water in a public swimming pool (5)
- in the forest (6)

| 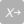 |
| --- |

Hygene_WashHands In which situations do you think people should wash their hands (tick any that apply)?

- before eating a meal (1)
- after eating a meal (2)
- after defecating (3)
- after urinating (4)
- when they come home (5)
- after shaking someone's hand (6)

End of Block: Hygiene norms

Start of Block: Privacy

| 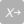 |
| --- |

Privacy_Online I think people should be more restrictive about disclosing information about themselves online

- Strongly agree (6)
- Moderately agree (5)
- Slightly agree (4)
- Slightly disagree (3)
- Moderately disagree (2)
- Strongly disagree (1)

| 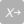 |
| --- |

Privacy_Gossip I think people should feel free to share information they have been told by friends and family, unless they have explicitly been asked to keep it secret

- Strongly agree (1)
- Moderately agree (2)
- Slightly agree (3)
- Slightly disagree (4)
- Moderately disagree (5)
- Strongly disagree (6)

| 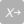 |
| --- |

Privacy_Secret I think it is rude to ask people to keep something a secret

- Strongly agree (1)
- Moderately agree (2)
- Slightly agree (3)
- Slightly disagree (4)
- Moderately disagree (5)
- Strongly disagree (6)

End of Block: Privacy

Start of Block: Gelfand's perceived tightness

Tightness_info The following statements refer to the country in which you live as a whole. Please indicate whether you agree or disagree with the following statements using the following scale. Note that the statements sometimes refer to "social norms,” which are standards for behavior that are generally unwritten.

| 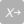 |
| --- |

Tightness_Q1 There are many social norms that people are supposed to abide by in this country

- Strongly agree (6)
- Moderately agree (5)
- Slightly agree (4)
- Slightly disagree (3)
- Moderately disagree (2)
- Strongly disagree (1)

| 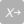 |
| --- |

Tightness_Q2 In this country, there are very clear expectations for how people should act in most situations

- Strongly agree (6)
- Moderately agree (5)
- Slightly agree (4)
- Slightly disagree (3)
- Moderately disagree (2)
- Strongly disagree (1)

| 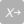 |
| --- |

Tightness_Q3 People agree upon what behaviors are appropriate versus inappropriate in most situations this country

- Strongly agree (6)
- Moderately agree (5)
- Slightly agree (4)
- Slightly disagree (3)
- Moderately disagree (2)
- Strongly disagree (1)

| 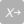 |
| --- |

Tightness_Q4 People in this country have a great deal of freedom in deciding how they want to behave in most situations

- Strongly agree (1)
- Moderately agree (2)
- Slightly agree (3)
- Slightly disagree (4)
- Moderately disagree (5)
- Strongly disagree (6)

| 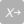 |
| --- |

Tightness_Q5 In this country, if someone acts in an inappropriate way, others will strongly disapprove

- Strongly agree (6)
- Moderately agree (5)
- Slightly agree (4)
- Slightly disagree (3)
- Moderately disagree (2)
- Strongly disagree (1)

| 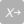 |
| --- |

Tightness_Q6 People in this country almost always comply with social norms

- Strongly agree (6)
- Moderately agree (5)
- Slightly agree (4)
- Slightly disagree (3)
- Moderately disagree (2)
- Strongly disagree (1)

End of Block: Gelfand's perceived tightness

Start of Block: Perceived threat to society

| 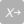 |
| --- |

Perceived_threat Which of the following do you think of as real threats to your society (tick *all* that apply)

- conflict within the country (1)
- conflict with other countries (2)
- over-population (3)
- food deprivation (4)
- lack of safe water (5)
- poor quality of air (6)
- natural disasters (7)
- diseases (8)
- immigration (9)

End of Block: Perceived threat to society

Start of Block: Animation - Stealing

AniSteal1 Click the play button to start the animation. It lasts for about 1 minute. You may watch it again from the beginning. Watch it to the end at least once before making your judgments.

AniSteal2 After the animation we will refer to the triangles by their colors. In case you cannot see colors, the triangle based in the top left corner is BLUE and the bottom right triangle is PURPLE.

AniSteal3   <https://www.youtube.com/watch?v=X8xA5ZDFA0s&feature=youtu.be>

| 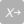 |
| --- |

StealApp How appropriate was it for the PURPLE triangle to behave as it did?

- extremely inappropriate (0)
- very inappropriate (1)
- somewhat inappropriate (2)
- somewhat appropriate (3)
- very appropriate (4)
- extremely appropriate (5)

StealFeel How do you feel about the PURPLE triangle's behavior? (check all that apply)

- happy (1)
- sad (2)
- surprised (3)
- afraid (4)
- disgusted (5)
- angry (6)
- satisfied (7)
- another positive emotion (8)
- another negative emotion (9)

StealABCD Suppose a person behaves like the PURPLE triangle did, and that this behavior is observed by persons A, B, C, and D - who react in different ways.

StealNoth **Person A does nothing about the person who behaved like the PURPLE triangle.**

| 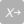 |
| --- |

StealNothApp How appropriate would it be for Person A to react in this way?

- extremely inappropriate (0)
- very inappropriate (1)
- somewhat inappropriate (2)
- somewhat appropriate (3)
- very appropriate (4)
- extremely appropriate (5)

StealRemark **Person B makes an angry remark to the person who behaved like the PURPLE triangle.**

| 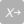 |
| --- |

StealRemarkApp How appropriate would it be for Person B to react in this way?

- extremely inappropriate (0)
- very inappropriate (1)
- somewhat inappropriate (2)
- somewhat appropriate (3)
- very appropriate (4)
- extremely appropriate (5)

StealTalk **Person C talks to someone else about the person who behaved like the PURPLE triangle.**

| 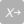 |
| --- |

StealTalkApp How appropriate would it be for Person C to react in this way?

- extremely inappropriate (0)
- very inappropriate (1)
- somewhat inappropriate (2)
- somewhat appropriate (3)
- very appropriate (4)
- extremely appropriate (5)

StealAvoid **Person D makes a point of avoiding the person who behaved like the PURPLE triangle in the future, even when that person is not behaving in this way.**

| 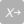 |
| --- |

StealAvoidApp How appropriate would it be for Person D to react in this way?

- extremely inappropriate (0)
- very inappropriate (1)
- somewhat inappropriate (2)
- somewhat appropriate (3)
- very appropriate (4)
- extremely appropriate (5)

End of Block: Animation - Stealing

Start of Block: Animation - Stealing Physical Punishment

AniStealPhysPun1 Watch the following animation of the BLUE triangle reacting to the PURPLE triangle's earlier behavior.

AniStealPhysPun2     <https://www.youtube.com/watch?v=wAkGLz_tWs0&feature=youtu.be>

| 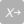 |
| --- |

StealPhysPunApp How appropriate was it for the BLUE triangle to behave as it did?

- extremely inappropriate (0)
- very inappropriate (1)
- somewhat inappropriate (2)
- somewhat appropriate (3)
- very appropriate (4)
- extremely appropriate (5)

StealPhysPunABCD Suppose a person behaves like the BLUE triangle did, and that this behavior is observed by persons A, B, C, and D - who react in different ways.

StealPhysPunRemark **Person B makes an angry remark to the person who behaved like the BLUE triangle.**

| 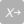 |
| --- |

StealPhysPunRemApp How appropriate would it be for Person B to react in this way?

- extremely inappropriate (0)
- very inappropriate (1)
- somewhat inappropriate (2)
- somewhat appropriate (3)
- very appropriate (4)
- extremely appropriate (5)

StealPhysPunTalk **Person C talks to someone else about the person who behaved like the BLUE triangle.**

| 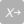 |
| --- |

StealPhysPunTalkApp How appropriate would it be for Person C to react in this way?

- extremely inappropriate (0)
- very inappropriate (1)
- somewhat inappropriate (2)
- somewhat appropriate (3)
- very appropriate (4)
- extremely appropriate (5)

StealPhysPunAvoid **Person D makes a point of avoiding the person who behaved like the BLUE triangle in the future, even when that person is not behaving in this way.**

| 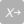 |
| --- |

StealPhysPunAvoidApp How appropriate would it be for Person D to react in this way?

- extremely inappropriate (0)
- very inappropriate (1)
- somewhat inappropriate (2)
- somewhat appropriate (3)
- very appropriate (4)
- extremely appropriate (5)

StealPhysPunNoth **Person A does nothing about the person who behaved like the BLUE triangle.**

| 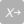 |
| --- |

StealPhysPunNothApp How appropriate would it be for Person A to react in this way?

- extremely inappropriate (0)
- very inappropriate (1)
- somewhat inappropriate (2)
- somewhat appropriate (3)
- very appropriate (4)
- extremely appropriate (5)

End of Block: Animation - Stealing Physical Punishment

Start of Block: Scenario 1

Sce1 Imagine a funeral. One of the guests (**Guest A**) wears headphones, apparently listening to music during the ceremony.

| 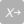 |
| --- |

Sce1AApp How appropriate is it for Guest A to listen to music on headphones at a funeral?

- extremely inappropriate (0)
- very inappropriate (1)
- somewhat inappropriate (2)
- somewhat appropriate (3)
- very appropriate (4)
- extremely appropriate (5)

Sce1AFeel How do you feel about Guest A's behavior? (check all that apply)

- happy (1)
- sad (2)
- surprised (3)
- afraid (4)
- disgusted (5)
- angry (6)
- satisfied (7)
- another positive emotion (8)
- another negative emotion (9)

Sce1BCDEInfo Guests B, C, D and E all think it is bad behavior to listen to music on headphones at a funeral, but they react in different ways.

Sce1CInfo **Guest C makes an angry remark to Guest A about the headphones.**

| 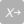 |
| --- |

Sce1CApp How appropriate would it be for Guest C to react in this way?

- extremely inappropriate (0)
- very inappropriate (1)
- somewhat inappropriate (2)
- somewhat appropriate (3)
- very appropriate (4)
- extremely appropriate (5)

Sce1EInfo **Guest E makes a point of avoiding Guest A in the future, even when Guest A is not listening to music on headphones at a funeral.**

| 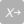 |
| --- |

Sce1EApp How appropriate would it be for Guest E to react in this way?

- extremely inappropriate (0)
- very inappropriate (1)
- somewhat inappropriate (2)
- somewhat appropriate (3)
- very appropriate (4)
- extremely appropriate (5)

Sce1BInfo **Guest B does nothing about Guest A.**

| 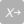 |
| --- |

Sce1BApp How appropriate would it be for Guest B to react in this way?

- extremely inappropriate (0)
- very inappropriate (1)
- somewhat inappropriate (2)
- somewhat appropriate (3)
- very appropriate (4)
- extremely appropriate (5)

Sce1DInfo **Guest D talks to someone else about Guest A listening to music on headphones at a funeral.**

| 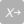 |
| --- |

Sce1DApp How appropriate would it be for Guest D to react in this way?

- extremely inappropriate (0)
- very inappropriate (1)
- somewhat inappropriate (2)
- somewhat appropriate (3)
- very appropriate (4)
- extremely appropriate (5)

End of Block: Scenario 1

Start of Block: Scenario 2

Sce2 Imagine a restaurant. One customer (**Customer A**) is sleeping at a table.

| 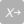 |
| --- |

Sce2AApp How appropriate is it for Customer A to sleep in a restaurant?

- extremely inappropriate (0)
- very inappropriate (1)
- somewhat inappropriate (2)
- somewhat appropriate (3)
- very appropriate (4)
- extremely appropriate (5)

Sce2AFeel How do you feel about Customer A's behavior? (check all that apply)

- happy (1)
- sad (2)
- surprised (3)
- afraid (4)
- disgusted (5)
- angry (6)
- satisfied (7)
- another positive emotion (8)
- another negative emotion (9)

Sce2BCDEInfo Customers B, C, D and E all think it is bad behavior to sleep at the table at a restaurant, but they react in different ways.

Sce2BInfo **Customer B does nothing about Customer A.**

| 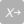 |
| --- |

Sce2BApp How appropriate would it be for Customer B to react in this way?

- extremely inappropriate (0)
- very inappropriate (1)
- somewhat inappropriate (2)
- somewhat appropriate (3)
- very appropriate (4)
- extremely appropriate (5)

Sce2DInfo
**Customer D talks to someone else about Customer A sleeping at the table.**

| 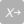 |
| --- |

Sce2DApp How appropriate would it be for Customer D to react in this way?

- extremely inappropriate (0)
- very inappropriate (1)
- somewhat inappropriate (2)
- somewhat appropriate (3)
- very appropriate (4)
- extremely appropriate (5)

Sce2BInfo **Customer C makes an angry remark to Customer A about sleeping at the table.**

| 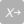 |
| --- |

Sce2CApp How appropriate would it be for Customer C to react in this way?

- extremely inappropriate (0)
- very inappropriate (1)
- somewhat inappropriate (2)
- somewhat appropriate (3)
- very appropriate (4)
- extremely appropriate (5)

Sce2EInfo **Customer E makes a point of avoiding Customer A in the future, even when Customer A is not sleeping at a table in a restaurant.**

| 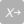 |
| --- |

Sce2EApp How appropriate would it be for Customer E to react in this way?

- extremely inappropriate (0)
- very inappropriate (1)
- somewhat inappropriate (2)
- somewhat appropriate (3)
- very appropriate (4)
- extremely appropriate (5)

End of Block: Scenario 2

Start of Block: Scenario 3

Sce3 Imagine a library. One visitor (**Visitor A**) starts singing.

| 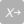 |
| --- |

Sce3AApp How appropriate is it for Visitor A to sing at a library?

- extremely inappropriate (0)
- very inappropriate (1)
- somewhat inappropriate (2)
- somewhat appropriate (3)
- very appropriate (4)
- extremely appropriate (5)

Sce3AFeel How do you feel about Visitor A's behavior? (check all that apply)

- happy (1)
- sad (2)
- surprised (3)
- afraid (4)
- disgusted (5)
- angry (6)
- satisfied (7)
- another positive emotion (8)
- another negative emotion (9)

Sce3BCDEInfo Visitors B, C, D and E all think it is bad behavior to sing at a library, but they react in different ways.

Sce3CInfo **Visitor C makes an angry remark to Visitor A about singing**.

| 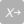 |
| --- |

Sce3CApp How appropriate would it be for Visitor C to react in this way?

- extremely inappropriate (0)
- very inappropriate (1)
- somewhat inappropriate (2)
- somewhat appropriate (3)
- very appropriate (4)
- extremely appropriate (5)

Sce3EInfo **Visitor E makes a point of avoiding Visitor A in the future, even when Visitor A is not singing at a library.**

| 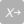 |
| --- |

Sce3EApp How appropriate would it be for Visitor E to react in this way?

- extremely inappropriate (0)
- very inappropriate (1)
- somewhat inappropriate (2)
- somewhat appropriate (3)
- very appropriate (4)
- extremely appropriate (5)

Sce3BInfo **Visitor B does nothing about Visitor A.**

| 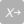 |
| --- |

Sce3BApp How appropriate would it be for Visitor B to react in this way?

- extremely inappropriate (0)
- very inappropriate (1)
- somewhat inappropriate (2)
- somewhat appropriate (3)
- very appropriate (4)
- extremely appropriate (5)

Sce3DInfo **Visitor D talks to someone else about Visitor A singing at the library.**

| 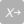 |
| --- |

Sce3DApp How appropriate would it be for Visitor D to react in this way?

- extremely inappropriate (0)
- very inappropriate (1)
- somewhat inappropriate (2)
- somewhat appropriate (3)
- very appropriate (4)
- extremely appropriate (5)

End of Block: Scenario 3

Start of Block: Scenario 4

Sce4 Imagine a movie theater where a movie is showing. One audience member (**Audience member A**) is reading the newspaper.

| 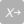 |
| --- |

Sce4AApp How appropriate is it for Audience member A to read in the movie theater while a movie is showing?

- extremely inappropriate (0)
- very inappropriate (1)
- somewhat inappropriate (2)
- somewhat appropriate (3)
- very appropriate (4)
- extremely appropriate (5)

Sce4AFeel How do you feel about Audience member A's behavior? (check all that apply)

- happy (1)
- sad (2)
- surprised (3)
- afraid (4)
- disgusted (5)
- angry (6)
- satisfied (7)
- another positive emotion (8)
- another negative emotion (9)

Sce4BCDEInfo Audience members B, C, D and E all think it is bad behavior to read in the movie theater, but they react in different ways.

Sce4EInfo **Audience member E makes a point of avoiding Audience member A in the future, even when Audience member A is not reading in a movie theater.**

| 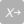 |
| --- |

Sce4EApp How appropriate would it be for Audience member E to react in this way?

- extremely inappropriate (0)
- very inappropriate (1)
- somewhat inappropriate (2)
- somewhat appropriate (3)
- very appropriate (4)
- extremely appropriate (5)

Sce4DInfo **Audience member D talks to someone else about Audience member A reading at the movie theater.**

| 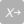 |
| --- |

Sce4DApp How appropriate would it be for Audience member D to react in this way?

- extremely inappropriate (0)
- very inappropriate (1)
- somewhat inappropriate (2)
- somewhat appropriate (3)
- very appropriate (4)
- extremely appropriate (5)

Sce4BInfo **Audience member B does nothing about Audience member A.**

| 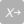 |
| --- |

Sce4BApp How appropriate would it be for Audience member B to react in this way?

- extremely inappropriate (0)
- very inappropriate (1)
- somewhat inappropriate (2)
- somewhat appropriate (3)
- very appropriate (4)
- extremely appropriate (5)

Sce4CInfo **Audience member C makes an angry remark to Audience member A about reading.**

| 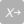 |
| --- |

Sce4CApp How appropriate would it be for Audience member C to react in this way?

- extremely inappropriate (0)
- very inappropriate (1)
- somewhat inappropriate (2)
- somewhat appropriate (3)
- very appropriate (4)
- extremely appropriate (5)

End of Block: Scenario 4

Start of Block: Insult

| 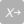 |
| --- |

InsultAppropriate Some men would respond with physical aggression if someone insulted their mother, other men would not. How appropriate do you think it is for men to use physical aggression when someone insults their mother?

- extremely inappropriate (0)
- very inappropriate (1)
- somewhat inappropriate (2)
- somewhat appropriate (3)
- very appropriate (4)
- extremely appropriate (5)

InsultFeel How do you feel about someone responding with physical aggression to an insult? (check all that apply)

- happy (1)
- sad (2)
- surprised (3)
- afraid (4)
- disgusted (5)
- angry (6)
- satisfied (7)
- another positive emotion (8)
- another negative emotion (9)

InsultBCDEInfo Person A responds with physical aggression when someone insulted his mother. This is observed by Persons B, C, D and E. They all think it is bad behavior to respond with physical aggression to such an insult, but they react in different ways.

InsultCInfo **Person C makes an angry remark to Person A about responding with physical aggression to the insult about his mother.**

| 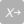 |
| --- |

InsultCApp How appropriate would it be for Person C to react in this way?

- extremely inappropriate (0)
- very inappropriate (1)
- somewhat inappropriate (2)
- somewhat appropriate (3)
- very appropriate (4)
- extremely appropriate (5)

InsultBInfo **Person B does nothing about Person A.**

| 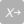 |
| --- |

InsultBApp How appropriate would it be for Person B to react in this way?

- extremely inappropriate (0)
- very inappropriate (1)
- somewhat inappropriate (2)
- somewhat appropriate (3)
- very appropriate (4)
- extremely appropriate (5)

InsultEHang **Person E makes a point of avoiding Person A in the future, even when Person A is not responding with physical aggression to an insult.**

| 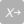 |
| --- |

InsultEApp How appropriate would it be for Person E to react in this way?

- extremely inappropriate (0)
- very inappropriate (1)
- somewhat inappropriate (2)
- somewhat appropriate (3)
- very appropriate (4)
- extremely appropriate (5)

InsultDInfo **Person D talks with someone else about Person A responding with physical aggression to the insult about his mother.**

| 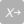 |
| --- |

InsultDApp How appropriate would it be for Person D to react in this way?

- extremely inappropriate (0)
- very inappropriate (1)
- somewhat inappropriate (2)
- somewhat appropriate (3)
- very appropriate (4)
- extremely appropriate (5)

End of Block: Insult

Start of Block: Meta-norm Reprimand

Reprimand **Imagine Person A reprimanding someone, who has been rude in a public place.**

| 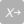 |
| --- |

ReprimandApp How appropriate would it be for Person A to react in this way?

- extremely inappropriate (0)
- very inappropriate (1)
- somewhat inappropriate (2)
- somewhat appropriate (3)
- very appropriate (4)
- extremely appropriate (5)

ReprimandFeel How do you feel about Person A's behavior? (check all that apply)

- happy (1)
- sad (2)
- surprised (3)
- afraid (4)
- disgusted (5)
- angry (6)
- satisfied (7)
- another positive emotion (8)
- another negative emotion (9)

ReprimandBCDE **Persons B, C, D and E all think it is bad behavior to reprimand someone, but they react in different ways.**

ReprimandTalk **Person D talks to someone else about Person A.**

| 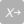 |
| --- |

ReprimandTalkApp How appropriate would it be for Person D to react in this way?

- extremely inappropriate (0)
- very inappropriate (1)
- somewhat inappropriate (2)
- somewhat appropriate (3)
- very appropriate (4)
- extremely appropriate (5)

ReprimandNothing **Person B does nothing about Person A.**

| 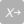 |
| --- |

ReprimandNothingApp How appropriate would it be for Person B to react in this way?

- extremely inappropriate (0)
- very inappropriate (1)
- somewhat inappropriate (2)
- somewhat appropriate (3)
- very appropriate (4)
- extremely appropriate (5)

ReprimandRemark **Person C makes an angry remark to Person A.**

| 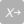 |
| --- |

ReprimandRemarkApp How appropriate would it be for Person C to react in this way?

- extremely inappropriate (0)
- very inappropriate (1)
- somewhat inappropriate (2)
- somewhat appropriate (3)
- very appropriate (4)
- extremely appropriate (5)

ReprimandAvoid **Person E makes a point of avoiding Person A, even when Person A is not behaving in this way.**

| 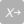 |
| --- |

ReprimandAvoidApp How appropriate would it be for Person E to react in this way?

- extremely inappropriate (0)
- very inappropriate (1)
- somewhat inappropriate (2)
- somewhat appropriate (3)
- very appropriate (4)
- extremely appropriate (5)

End of Block: Meta-norm Reprimand

Start of Block: Meta-norm Speaking Negatively

SpeakNeg **Imagine Person A speaking negatively (to another person) about someone who has been rude in a public place.**

| 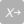 |
| --- |

SpeakNegApp How appropriate would it be for Person A to react in this way?

- extremely inappropriate (0)
- very inappropriate (1)
- somewhat inappropriate (2)
- somewhat appropriate (3)
- very appropriate (4)
- extremely appropriate (5)

SpeakNegFeel How do you feel about Person A's behavior? (check all that apply)

- happy (1)
- sad (2)
- surprised (3)
- afraid (4)
- disgusted (5)
- angry (6)
- satisfied (7)
- another positive emotion (8)
- another negative emotion (9)

SpeakNegBCDE **Persons B, C, D and E all think it is bad behavior to speak negatively about someone, but they react in different ways.**

SpeakNegRemark **Person C makes an angry remark to Person A.**

| 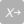 |
| --- |

SpeakNegRemarkApp How appropriate would it be for Person C to react in this way?

- extremely inappropriate (0)
- very inappropriate (1)
- somewhat inappropriate (2)
- somewhat appropriate (3)
- very appropriate (4)
- extremely appropriate (5)

SpeakNegAvoid **Person E makes a point of avoiding Person A, even when Person A is not behaving in this way.**

| 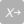 |
| --- |

SpeakNegAvoidApp How appropriate would it be for Person E to react in this way?

- extremely inappropriate (0)
- very inappropriate (1)
- somewhat inappropriate (2)
- somewhat appropriate (3)
- very appropriate (4)
- extremely appropriate (5)

SpeakNegNothing **Person B does nothing about Person A.**

| 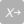 |
| --- |

SpeakNegNothingApp How appropriate would it be for Person B to react in this way?

- extremely inappropriate (0)
- very inappropriate (1)
- somewhat inappropriate (2)
- somewhat appropriate (3)
- very appropriate (4)
- extremely appropriate (5)

SpeakNegTalk Person D talks to someone else about Person A.

| 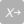 |
| --- |

SpeakNegTalkApp How appropriate would it be for Person D to react in this way?

- extremely inappropriate (0)
- very inappropriate (1)
- somewhat inappropriate (2)
- somewhat appropriate (3)
- very appropriate (4)
- extremely appropriate (5)

End of Block: Meta-norm Speaking Negatively

Start of Block: Meta-norm Stay Away

StayAway **Imagine Person A staying away from someone who has been rude in a public place.**

| 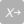 |
| --- |

StayAwayApp How appropriate would it be for Person A to react in this way?

- extremely inappropriate (0)
- very inappropriate (1)
- somewhat inappropriate (2)
- somewhat appropriate (3)
- very appropriate (4)
- extremely appropriate (5)

StayAwayFeel How do you feel about Person A's behavior? (check all that apply)

- happy (1)
- sad (2)
- surprised (3)
- afraid (4)
- disgusted (5)
- angry (6)
- satisfied (7)
- another positive emotion (8)
- another negative emotion (9)

StayAwayApp **Persons B, C, D and E all think it is bad behavior to stay away from someone, but they react in different ways.**

StayAwayNothing **Person B does nothing about Person A.**

| 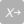 |
| --- |

StayAwayNothingApp How appropriate would it be for Person B to react in this way?

- extremely inappropriate (0)
- very inappropriate (1)
- somewhat inappropriate (2)
- somewhat appropriate (3)
- very appropriate (4)
- extremely appropriate (5)

StayAwayRemark **Person C makes an angry remark to Person A.**

| 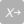 |
| --- |

StayAwayRemarkApp How appropriate would it be for Person C to react in this way?

- extremely inappropriate (0)
- very inappropriate (1)
- somewhat inappropriate (2)
- somewhat appropriate (3)
- very appropriate (4)
- extremely appropriate (5)

StayAwayAvoid **Person E makes a point of avoiding Person A, even when Person A is not behaving in this way.**

| 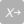 |
| --- |

StayAwayAvoidApp How appropriate would it be for Person E to react in this way?

- extremely inappropriate (0)
- very inappropriate (1)
- somewhat inappropriate (2)
- somewhat appropriate (3)
- very appropriate (4)
- extremely appropriate (5)

StayAwayTalk Person D talks to someone else about Person A.

| 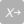 |
| --- |

StayAwayTalkApp How appropriate would it be for Person D to react in this way?

- extremely inappropriate (0)
- very inappropriate (1)
- somewhat inappropriate (2)
- somewhat appropriate (3)
- very appropriate (4)
- extremely appropriate (5)

End of Block: Meta-norm Stay Away

Start of Block: Demographics

Background Thank you for completing the animation and the scenarios. You are almost done.

Gender What is your gender?

- Male (1)
- Female (2)
- Other/Don't want to say (3)

| 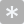 |
| --- |

Age What is your age?

________________________________________________________________

Country In which country do you currently reside?

________________________________________________________________

Origin Did you grow up in the same country as the one that you are currently living in?

- Yes (1)
- No (2)

| 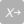 |
| --- |

Degree What is the highest level of school your most educated parent has completed, or the highest degree they have received?

- Less than high school degree (1)
- High school graduate (2)
- Some college but no degree (3)
- Bachelor's/Associate degree (4)
- Master's degree (5)
- Doctoral degree (6)

| 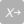 |
| --- |

Major What is your academic focus or major?

- Economics (1)
- Psychology (1)
- Other (3)
- Undecided (4)
- I am not a student (5)

End of Block: Demographics

Start of Block: Attention check

Attention This is an attention check, please choose 4.

- 1 (1)
- 2 (2)
- 3 (3)
- 4 (4)
- 5 (5)

End of Block: Attention check

Start of Block: Comments

Comment This is a study of cultural differences in reactions to possible norm violations. Please provide any comments you may have below, we appreciate your input on the study!

________________________________________________________________

________________________________________________________________

________________________________________________________________

________________________________________________________________

________________________________________________________________

| 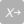 |
| --- |

Comprehension Was it easy or difficult for you to understand the questions in this survey?

- very easy (5)
- easy (4)
- neither easy nor difficult (3)
- difficult (2)
- very difficult (1)

End of Block: Comments

Start of Block: Finish

Thanks Thank you for completing the survey!

Code Here is your participation code:

Code ${e://Field/Random%20ID}

Responsible *Responsible for this study is professor Kimmo Eriksson, Stockholm University.*

End of Block: Finish
