# Supplementary material for: Global sex differences in hygiene norms and their relation to sex equality
Source: PLOS Glob Public Health. 2022 Jun 21;2(6):e0000591. doi: 10.1371/journal.pgph.0000591 (PMC10021886; doi:10.1371/journal.pgph.0000591)
Supplement: Attachment — Submitted filename: Rebuttal R1.docx [file pgph.0000591.s006.docx]

We are very happy with the invitation to revise and resubmit our manuscript PGPH-D-21-00958 “Global sex differences in hygiene norms and their relation to sex equality” for publication in PLOS Global Public Health. Here is how we have addressed the comments from the editor and reviewers.

**Comments from the editor**

1. Please include additional information regarding the survey or questionnaire used in the study and ensure that you have provided sufficient details that others could replicate the analyses. For instance, if you developed a questionnaire as part of this study and it is not under a copyright more restrictive than CC-BY, please include a copy, in both the original language and English, as Supporting Information.

AUTHORS: The full survey is now included as “S1 File”.

2. Please amend your detailed Financial Disclosure statement. This is published with the article, therefore should be completed in full sentences and contain the exact wording you wish to be published.

State what role the funders took in the study. If the funders had no role in your study, please state: “The funders had no role in study design, data collection and analysis, decision to publish, or preparation of the manuscript.”

AUTHORS: The statement now reads “Kimmo Eriksson acknowledges financial support from the Swedish Foundation for Humanities and Social Sciences (Riksbankens Jubileumsfond) [grant no. P17-0030:1]. Pontus Strimling acknowledges financial support from the Knut and Alice Wallenberg Foundation [grant no. 2017.0257]. The funders had no role in study design, data collection and analysis, decision to publish, or preparation of the manuscript.”

3. Please note that Figure 1 is currently split into multiple parts in separate files. Please either combine the parts into one figure with lettered panels, or renumber your figures as whole parts (i.e. Fig 1A becomes Fig 1, Fig 1B becomes Fig 2 etc.), ensuring that all relevant legends and in-text citations have been updated accordingly.

AUTHORS: We have renumbered the figures accordingly.

**Comments from Reviewer #1**

Global sex difference in hygiene norms and their relation to sex equality is a noble study, given the role of hygiene in dairrheal and other infectious diseases spread worldwide.

This study demonstrate sex differences in terms of stricter hygiene norms; however, it would be of interest to also look at some of the explanatory variables for this observed universal sex difference in hygiene strictness in a large cross-cultural sample.

What is not clear, the sample size distribution per country was based on? an whether they would have an influence on the statistical outcome.

AUTHORS: Thank you. We have clarified the sampling goal of the ISMN as follows: “Hygiene norms were measured in 56 countries as part of ISMN, the International Study of Metanorms (8,17). This study aimed at collecting samples of at least 200 students per country and, in most countries, an additional 100 non-students. In the present study of sex differences we include only those participants who also reported information on sex, yielding final sample sizes per country ranging from 45 to 1,009 with a median of 281”. With regards to the statistical outcome, we note that the individual-level analysis (Table 2) shows that the main findings are statistically significant while the scatterplots illustrate that the non-linear effect is clear to the naked eye.

**Comments from Reviewer #2**

This paper describes data collected by the International Study of Metanorms project. In this study, the authors and their collaborators assessed the endorsements of a variety of norms across 56 nations. Relevant to the current paper, some of these norms concerned acts that can transmit pathogens – specifically, handwashing and spitting. The current manuscript reports that, across cultures, women were more likely (1) to find it inappropriate to spit and (2) to believe that people should wash their hands. A nation-level variable assessing endorsements of sex equality moderated this sex difference in a non-linear fashion. I think that this is a thought-provoking paper that would contribute nicely to multiple literatures. However, I think that some of the analyses should be better justified and potentially redone. I’ll provide a list of suggestions below.

ISSUE 1: OPERATIONALIZTION OF SEX EQUALITY.

The authors refer to sex equity in the manuscript title, abstract, and text, and they also refer to egalitarianism. But the variable they use to operationalize this construct assesses attitudes toward sex equality among citizens of those countries, as estimated from the World Values Survey (e.g., “University is more important for a boy than a girl”). This decision is justified as follows: “When examining causal effects of sex equality, it is preferable to use a belief measure instead of an outcome measure such as the Global Gender Gap Index; as other researchers has (sic) pointed out, the same outcome may be achieved in different ways.” I’m not persuaded by this justification, and I doubt that the majority of readers will be. After all, the argument presented in the introduction doesn’t concern attitudes; it concerns structural differences in the roles that the sexes typically fill. Ultimately, there’s little harm in also reporting how a more objective measure of sex equality moderates the sex differences reported in the manuscript. Differences in outcomes across the moderators could be useful in generating further hypotheses explaining these sex differences. I’d also prefer for the variables to be labeled in a more precise manner. For example, the WVS measure could be labeled “Sexist Attitudes,” whereas the Global Gender Index could be labeled “Sex Equality.”

AUTHORS: Thank you, we understand where the reviewer is coming from, although we do not fully agree. We believe that the cultural view of whether men and women are equal and should be treated equally is the best available measure of the sex roles of a society. In the Discussion, we discuss how moderately inegalitarian societies will prescribe different responsibilities to men and women in family life while the most inegalitarian societies may not view women as fully responsible for anything, having men as guardians. Ideally, we would have measures that directly taps into responsibilities in family life. Absent such measures, we think that the WVS measure of cultural valuations of sex equality is much closer to the target than the Global Gender Gap Index. As discussed in the study we cite, gender gaps in outcomes do not tell us whether they are driven by sex inequality (e.g., in countries that are inegalitarian but poor, women’s participation in the work force may still be high out of necessity). For these reasons, we have chosen to retain the focus on the WVS measure in the main text (and its label as “Sex equality”). We have included the scatter plots when the WVS measures is replaced by the GGGI as supplementary figures, while a supplementary table presents the corresponding results from individual-level analysis. The GGGI is a weaker predictor but otherwise the overall pattern is the same as the one we report in the article. The Methods section on this topic now reads:

“The level of sex equality in a country can be operationalized in various ways. We are concerned with sex inequality in terms of ingrained differences in the treatment of men and women solely due to their sex. The best available measure of this, we believe, is measures of cultural values with respect to treating men and women equally or differently. As detailed below, the World Values Survey provides such a measure (19). This is our key independent variable. An alternative operationalization of sex equality is the Global Gender Gap Index (GGGI), which estimates national gaps in outcomes between the group of men and the group of women with respect to economic participation and wages, educational attainment, political empowerment, and health and survival. It has been argued that different levels of structural sex equality may result in the same outcome and therefore that the GGGI is unsuitable to represent sex equality as a causal factor (20). Indeed, when recent research on sex differences in student achievement used both cultural values with respect to sex equality and the GGGI as independent predictors, only cultural values had an independent effect (21). In the current study, we therefore operationalize sex equality by cultural values. (In complementary analyses we used the GGGI instead. As expected, sex differences in hygiene are not as strongly predicted by the GGGI as by the cultural valuation of sex equality but the overall pattern of results is the same. See S1 Fig, S2 Fig, and S2 Table.)”

ISSUE 2: DATA AGGREGATION

I found the description of data aggregation (lines 237-252) confusing. The manuscript states “The internal consistency of the sex difference in the six handwashing norms is adequate (alpha = .78).” These analyses are either reported imprecisely, or they reflect an assessment of reliability that I’m unfamiliar with (and that departs markedly from standard practices). Coefficient alpha is based on the number of items and the average covariances between items, and it is an estimate of the proportion of observed variance attributable to variance in the construct of interest. A researcher might be interested in assessing the reliability of six handwashing norm items; this reliability would be computed as: (1) the number of items times the average interitem correlation divided by (2) one plus N minus 1 times the average interitem correlation. Estimating reliability in this manner and assigning each participant a single value of handwashing norm endorsement is a reasonable strategy for both reducing the observations that need to be analyzed and obtaining an estimate that less composed of idiosyncratic item-level variance. But the phrase “internal consistency of the sex difference” is puzzling. I’m not sure if the phrasing is imprecise, or if the analyses have been conducted at the national level, with each nation having an average sex difference across participants per item. This latter approach would be a problem. Variables can have similar sex differences at an aggregate level, but be uncorrelated at a lower level (this is analogous to the ecological fallacy). In sum, I’d urge either increased precision in the language used to describe these reliability estimates, or I’d urge reliability to be assessed at the item-level rather than at the country-level. (One other related note on this point: the authors have usefully included analysis script on the OSF, but the script for these reliability analyses was not included. I’d urge them to include this script, just to avoid confusion from other readers)

AUTHORS: In the cited paragraph we are assessing the internal consistency (across contexts) of national sex differences in norms about handwashing norms and spitting. Individual-level consistency is not the issue here. The point of this analysis is specifically to examine whether it is meaningful to aggregate national sex differences across contexts to reduce noise. This is meaningful precisely if there is a strong common pattern in how these national sex differences vary across countries. Cronbach’s alpha is the standard method to examine whether there is a strong common pattern of variation across a set of measures. Just as the reviewer points out, it is possible for correlational patterns to be different across levels of analysis. For this reason, establishing consistency at the individual level would not serve the purpose of this analysis. We have tried to clarify what we are doing by adding the following sentences:

“A way to reduce the noise is to aggregate national sex differences in hygiene norms across contexts. However, aggregation across contexts is a meaningful way to reduce noise only if there is a consistent underlying pattern in national sex differences in hygiene norms. We therefore first need to check that national sex differences in hygiene norms in different contexts indeed exhibit a sufficient level of consistency.”

The reviewer’s comment was also very useful in that in the reporting of the subsequent individual-level analysis we had forgotten to define the dependent variable at the individual level. We have now added the following information to that section:

“To mirror the country-level analysis, we average the individual’s dichotomous responses to handwashing in five contexts, ⍺ = .51. We similarly average the individual’s dichotomous responses to spitting in five contexts, ⍺ = .58. (These values of Cronbach’s alpha are on the low side, which tends to be the case when data are dichotomous.)”

ISSUE 3: TREATMENT OF SAUDI ARABIA

The findings from Saudi Arabia are fascinating. But I’d urge the authors to do two things. First, double check the data. In a past project, I organized data collection across 30 nations. The mean for one of the critical variables in this study was higher for women than for men in 28 of the 30 nations (more about this variable below). The sex difference was almost identical in magnitude, but in the opposite direction, for the other two nations. When I asked the researchers from those two nations to verify that they had coded for participant sex in the correct manner, they realized that they had made a mistake. Hence, the reversed sex difference was caused by a coding error.

Second, if the pattern remains after verifying that the data are correct, I’d encourage a bit more reflection on why that pattern might exist. Consider, for example, the role of hygiene (especially handwashing) in Islamic prayer rituals (e.g., Wadu). Ritualized hygiene practices might attenuate the sex difference; ritualized hygiene practices combined with some sex segregation in these practices might reverse them. Naturally, these thoughts are speculative. Nevertheless, speculation can lay the ground for future research.

AUTHORS: Thanks for good suggestions! We have double-checked the Qualtrics survey, and gender is coded correctly in Saudi Arabia and also in Nigeria. (A coding error would have been very surprising given that, as we discuss, several previous studies of hygiene in Saudi Arabia have found the same reversed sex effect there.) We have expanded our reflection a little bit as follows.

“As a case in point, all women in the extremely unequal society of Saudi Arabia have a legal male guardian who is responsible for them (27). Among other things, this ultra-low level of women’s responsibility means a lower level of responsibility for hygiene. Sex segregation in Saudi Arabia also implies that women are allowed less mobility and thereby potentially less exposure to situations (including religious rituals) that may motivate hygiene norms. This could be examined in future research.”

We did not specifically mention the ritualized handwashing here because we don’t think a sufficiently credible case can be made for it. For one thing, women too engage in some ritualized handwashing. For another, our first paper on this dataset shows that Saudi Arabia has among the loosest hygiene norms in the world, which suggests that ritualized handwashing is not an important cause of stricter hygiene norms.

ISSUE 4: LASER FOCUS ON SOCIAL ROLES

As noted earlier, I think that this work is very interesting, and I suspect that others will feel the same. I do think it could be a bit broader, though. Consider the project I alluded to above (<http://www.pnas.org/cgi/doi/10.1073/pnas.1607398113>). We assessed pathogen disgust sensitivity across 30 nations. That is, we asked participants to report how disgusting they find stepping on dog poop, seeing mold on food, sitting close to someone with red sores on their arms, etc. The mean for women was higher than the mean for men in every one of these 30 nations. Now, perhaps this sex difference also emerges from the systemic differences in social roles across the sexes. Though I doubt it. A large body of work has mostly not supported the idea that such structural differences explain sex differences in personality, mate preferences, occupational goals, etc. See DOI:10.1007/978-3-319-09384-0_11 Table 11.1 for a succinct summary of these efforts. Ultimately, the structural hypotheses that the authors test are definitely worth testing! But they’re not the only game in town for explaining sex differences. And there’s a lot of work assessing and explaining sex differences in emotions and behaviors that neutralize pathogens. Some greater engagement with this work might strengthen the manuscript. I’ll provide a few further references to consider.

<https://doi.org/10.1007/978-1-4939-0314-6_15>

<https://doi.org/10.1177/1754073917709940>

<https://doi.org/10.1016/j.paid.2011.04.003>

I hope that these comments are useful, and I wish the authors the best.

AUTHORS: Thank you for pointing us to these sources. They helped us to clarify some points and inspired us to add an additional analysis to show the unique role that sex equality appears to play for sex differences in hygiene norms. In the Introduction we have added:

“The literature on sex differences has been divided between arguments for biological and social causes (Schmitt 2014). Within this literature disgust sensitivity has been inspected, with women repeatedly demonstrating greater disgust sensitivity than men (Fleischman 2014; Tybur et al. 2011; Al-Shawaf, Lewis, and Buss 2018). Disgust responses are not unrelated to hygiene behaviors and are thought to protect individuals from risky exposures that will be detrimental to average lifetime inclusive fitness. The sex difference in sensitivity has been attributed to asymmetries in fitness costs across the sexes, for example linking the female response to factors such as the unique challenges of pregnancy and post-partum obligate care (Fleischman 2014). Sex differences in disgust cannot fully explain the emergence of sex differences in generic normative standards for two reasons. First, hygiene practices and hence hygiene beliefs are not all directly tied to perceptible stimuli that elicit disgust. This is particularly the case for norms around handwashing, which is a core measure in the current study. Second, while the propensity for having normative beliefs about what ought to be done is not historically recent and we might anticipate biological limits on the *types* of beliefs that can be held, changes in their content, in the *tokens* of belief, can only have been caused by external contingencies. Hygiene practices have developed in recent historical time and represent a change in external contingency. If disgust response, or any other biologically caused disposition plays a role, those aspects of the behavioral phenotype have nonetheless interacted with new and emergent aspects of the relevant social ecology (Schmitt 2014). A starting point for investigation would be to explore cultural variation in sex differences in normative beliefs.”

In the Methods section:

“Alternative moderators. Inspired by Schmitt (16), we also examine whether variation across countries in the sex difference in hygiene norms may be driven by country differences in pathogen prevalence or religiosity rather than by sex equality. We used publicly available country-level data on the historical prevalence of pathogens (28) and religiosity (29).”

In the Results section:

“Alternative moderators. Finally, we examine whether country variation in sex differences in hygiene norms could be explained by pathogen prevalence or religiosity instead of sex equality (Schmitt 2014). To account for non-linearity in a comparable way across moderators, we use linear regression including a quadratic term in addition to a linear term. In such quadratic models, sex equality accounts for large proportions of the variation in national sex differences in hygiene strictness: 46% in the case of handwashing and 48% in the case of spitting. The alternative moderators fare much less well. Quadratic models that use religiosity instead only accounts for 5% and 14% of the variation, respectively. Quadratic models that use pathogen prevalence instead only accounts for 5% and 2% of the variation, respectively.”

In the Discussion:

“It is possible that spitting elicits a stronger disgust response in women by association because spitting is an innate behavioral response to remove noxious material from the mouth (Hennig, Pössel, and Netter 1996).”

and

“The sex difference in hygiene strictness was often larger in countries with above-average levels of sex equality than in countries with below-average levels of sex equality. This finding is in keeping with many behavioral and somatic sex difference results and Schmitt has argued that biological sex differences can be moderated and facilitated by specific cultural contexts (Schmitt 2014). To our knowledge it has not been shown for the contents of normative beliefs.”

and

“In countries with greater inequality, the sex difference in hygiene strictness disappeared and even became reversed at extreme levels of inequality (Saudi Arabia and Nigeria). This reversal suggests a flexible connection, if any, between hygiene norms and established sex differences in disgust sensitivity. But it is possible that the underlying asymmetry in inclusive fitness costs is something that can drive either male or female custodianship of hygiene, and hence difference in hygiene strictness, dependent upon key social ecological factors. Our analyses strongly supported that sex equality is a key factor. Specifically, while prior research has found that pathogen prevalence and religiosity may be more important factors behind sex differences in other domains (16), we found the level of sex equality do be a much stronger predictor of sex differences in hygiene.”
