# Supplementary material for: Global sex differences in hygiene norms and their relation to sex equality
Source: PLOS Glob Public Health. 2022 Jun 21;2(6):e0000591. doi: 10.1371/journal.pgph.0000591 (PMC10021886; doi:10.1371/journal.pgph.0000591)
Supplement: Attachment — Submitted filename: Rebuttal.R2.docx [file pgph.0000591.s007.docx]

I think that the authors have done a very nice job of revising their manuscript, and I endorse its publication. However, I think that the manuscript still contains a small but important problem, and it still could do a better job of integrating with the cross-cultural disgust literature. The second issue is one of preference on their part; the first issue, I think, is something that is simply wrong and should be amended for their sake.

First issue: the continued reporting of alpha of sex differences. Alpha is used to assess the reliability of a group of items. It's based on assumptions that each item in a group of items loads identically on a latent construct, and it estimates the degree to which the sum of those items reflects latent variable variance (relative to non-systematic or error variance). The construct of interest here is hygiene behaviors, and the authors are examining sex differences in that construct (and, further, seeing how those sex differences vary across a number of countries). Reporting alpha in the way that the manuscript continues to do is a problem for two reasons. First, by taking national averages, it removes information regarding individual-level responses. Second, and relatedly, by examining sex differences, it removes any information about how the items correlate with each other. Variables can have similar sex differences without relating to each other within the sexes. Again, please refer back to myriad examples of the ecological fallacy.

This is a straightforward issue to correct. Just report individual-level alphas (and, ideally, do so separately for each country in the supplement). The lower alpha at the individual level isn't necessarily a problem - it simply informs the degree to which the composites are noisy. I really do urge the authors to follow this suggestion - I think they're making a serious error by using alpha in the way that they use it, and they risk becoming yet another example of myriad inappropriate uses of coefficient alpha.

RESPONSE: We appreciate that the reviewer seeks to let us avoid making a serious error. Specifically the reviewer worries about possible ecological fallacies. An ecological fallacy usually means using a result obtained in a group level analysis to draw inferences about an individual level phenomenon. As this is not what is going on here, there is no ecological fallacy and in fact no error at all. It is not the case that it is “wrong” to calculate alpha scores for country-level data of sex differences in ratings of hygiene across items and “right” to calculate alpha scores for individual-level data on ratings of hygiene across items. The two analyses answer different questions and we perform the analysis that is relevant to our question. This is now clearly described in our paper:

“For every norm there are some countries in which the observed national sex difference is negative, that is, where men had somewhat stricter norms than women. However, for the clear majority of countries we observe a sex difference in strictness in the expected direction. Some of the aberrant results are likely due to noise. A way to reduce the noise is to aggregate national sex differences in hygiene norms across contexts. However, aggregation across contexts is a meaningful way to reduce noise only if there is a consistent underlying pattern in national sex differences in hygiene norms. We therefore first need to check that national sex differences in hygiene norms in different contexts indeed exhibit a sufficient level of consistency. The internal consistency of the national sex difference in the six handwashing norms is adequate, ⍺ = .78, indicating that it is meaningful to aggregate national sex differences in handwashing norms across contexts. (Note that this is a separate question from whether it is meaningful to aggregate individual ratings across contexts, whic is addressed in the mixed-level analysis below.)”

We hope this text makes it clear that our country-level alpha for sex differences is not used for any inferences about individual level consistency. It seems the reason behind the confusion is that the reviewer believes that “The construct of interest here is hygiene behaviors”. If this had been the case and our objective had been to show cross-item consistency of individual ratings, calculating alpha on individual-level data would have been the correct analysis. But our research question concerns *sex differences*, which are by definition group-level phenomena. A single individual cannot yield a sex difference measure. Cross-item consistency of sex differences, as measured by alpha, is what allows us to talk about a country’s sex difference in hygiene without having to specify the item.

Here is a thought experiment that may help. Imagine that ratings of spitting in different contexts are almost completely independent at the individual level (e.g., individual ratings might be determined by intelligence in one context, body size in another context, ideology in a third context, etc.). The only consistency is that there is a small sex difference that favors women in every context and whose size depend only on the country and not on the context. In this world, it would not make sense to aggregate individual ratings across items because we would lose an absurd amount of information about individual differences. In support of this conclusion, the individual-level alpha would be basically zero. But our study concern sex differences, we aggregate across all men and all women in a country and calculate the sex difference. In the imaginary world, we would observe that the country-differences in the sex difference are indeed consistent across items. The country-level alpha would be close to one. We would then draw the correct conclusion that the country effect on sex differences is context-independent, that is, we can conceive of there being a single latent country-level variable representing the sex difference in hygiene. To study this latent variable we would therefore be justified in aggregating sex differences across contexts to reduce measurement errors. This is exactly the use of alpha in our paper.

If the reviewer is still in doubt, here is a formal model. Cronbach’s alpha is the equivalent to the ICC for consistency of average measures, see McGraw & Wong (1996). When applied to the individual level, this means that Cronbach’s alpha measures how much of the total variance in individual ratings of many items (ignoring variance across items, that is, after removing the mean for each item) that is explained by the variance of a single individual-level latent variable. After the mean for each item is removed, the generating model for data is assumed to be

(1)	x_ij = r_i + e_ij,

where r_i is the individual-level latent variable and e_ij is the item-specific residual, both with zero mean. As these variables are not observable, we estimate r_i by x_i (the average of the x_ij across items) and the residuals by the differences of each rating to their average (x_ij – x_i). Cronbach’s alpha is obtained as 1 minus the ratio of the mean-square of the residuals to the mean-square of the x_i, which produces an unbiased estimate of the proportion of the total variance that is explained by the latent variable r_i.

Now we apply Cronbach’s alpha to country-level sex differences instead. The generating model at the individual level looks the same with two amendments: we need to include a country-dependent fixed effect of sex as well as a random intercept by country for each item (r_jc) with zero mean. A straightforward way is to write up one model for men:

(2)	xm_ijc = r_ic + r_jc + e_ijc

and another model for women that additionally includes the latent fixed effect of being female in a given country (F_c) plus any item-specific sex effect (f_jc):

(3)	xf_ijc = r_ic + r_jc + F_c + f_jc + e_ijc

Upon aggregation across all individuals in a country sample, equations (2) and (3) become

(4)	xm_jc = r_jc + em_jc

and

(5)	xf_jc = r_jc + F_c + f_jc + ef_jc

for women. In (4), xm_jc refers to the mean of all xm_ijc in country sample c and em_jc is the sampling error (the deviation between the actual sample mean of the residuals and the expected mean of zero). Similarly in (5), xf_jc refers to the mean of all xf_ijc in country sample c and ef_jc is the sampling error. We estimate the sex difference in a country as the difference between equations (5) and (4), that is,

(6)	sexdiff_jc = F_c + e_jc,

where e_jc is shorthand for the sum of three variables: f_jc + ef_jc – em_jc. Note that country-level equation (6) is, in mathematical terms, exactly analogous to the individual-level equation (1). Following this analody, we now approximate F_c by sexdiff_c (the average of sexdiff_jc across items) and estimate the residuals by (sexdiff_jc – sexdiff_c). Cronbach’s alpha applied to these data will tell us how much of the total variance in observed sex differences is explained by the latent variable F_c.

The crucial point is that the individual-level latent variable (r_ic) and the country-level variable (F_c) are entirely different variables. As our research question concerns the latter, the analysis must be chosen accordingly.

Second issue: I appreciated the inclusion of pathogen prevalence and some comments on disgust. I do think that the authors could say a bit more about these topics and link their paper with findings from this paper (<https://doi.org/10.1073/pnas.1607398113>), which (1) demonstrates cross-culturally stable sex differences in disgust (something that none of the papers currently cited on line 71 do, though the manuscript currently implies that they do), and (2) similarly finds a weak-to-null relation with pathogen prevalence (though the the paper I'm referring to examines pathogen disgust across the sexes rather than the sex difference). Again, this is the authors' choice. But I'd think that linking the current cross-cultural paper on hygiene norms to this cross-cultural paper on disgust (and its data on sex differences) would strengthen their contribution.

RESPONSE: Thank you for alerting us to this paper! We have added it in our reference list as number 20 and have amended the relevant part of the introduction to read as follows: “Within this literature disgust sensitivity has been inspected, with women repeatedly demonstrating greater disgust sensitivity than men (17-20). Disgust responses are not unrelated to hygiene behaviors and are thought to protect individuals from risky exposures that will be detrimental to average lifetime inclusive fitness, even if country measures of disgust sensitivity are not strongly related to pathogen prevalence (20).”
